# Robinow Syndrome: A Rare Case Report and Review of Literature

**DOI:** 10.5005/jp-journals-10005-1303

**Published:** 2015-08-11

**Authors:** Cristalle Soman, Ashok Lingappa

**Affiliations:** Assistant Professor, Department of Oral Medicine and Radiology, Amrita School of Dentistry, Kochi, Kerala, India; Professor and Head, Department of Oral Medicine and Radiology, Bapuji Dental College and Hospital, Davangere, Karnataka, India

**Keywords:** Alveolar ridge hypertrophy, Clinodactyly, Partial ankyloglossia, Robinow syndrome, Vertebral defects.

## Abstract

Robinow syndrome is an extremely rare genetic disorder. Short-limbed dwarfism, abnormalities in the head, face, and external genitalia, as well as vertebral defects comprise its distinct features. This disorder exists in dominant and recessive patterns. Patients with the dominant pattern exhibit moderate symptoms. More physical characteristics and skeletal abnormalities characterize the recessive group. The syndrome is also known as Robinow-Silverman-Smith syndrome, Robinow dwarfism, fetal face, fetal face syndrome, fetal facies syndrome, acral dysostosis with facial and genital abnormalities, or mesomelic dwarfism-small genitalia syndrome. Covesdem syndrome was the name entitled for the recessive form previously. Here, we report a case of 8-year-old female with a autosomal recessive Robinow syndrome having skeletal and vertebral defects.

**How to cite this article:** Soman C, Lingappa A. Robinow Syndrome: A Rare Case Report and Review of Literature. Int J Clin Pediatr Dent 2015;8(2):149-152.

## CASE REPORT

An 8-year-old female patient was accompanied by her father to the Department of Oral Medicine and Radiology, Bapuji Dental College and Hospital, Davangere, complaining of non-eruption of patients’ upper front tooth since 6 to 7 months. The father gives history of upper front milk teeth being fallen off approximately 6 to 7 months back due to mobility. There was no previous history of trauma. Past medical history revealed that the patient was consulted with a dentist approximately 1 year back with the concern of esthetic appearance of the patient, where there were given no treatment for the same. Family history revealed consanguineous marriage between parents and had three sibling; two elder sisters 15 and 12 years old and a younger brother 5 years old who were apparently normal but short in height. Personal history revealed that the patient used to brush her teeth once daily in the morning and was mixed in diet and have attained her milestones in accordance.

On general physical examination, the patient was found to present with short stature with shortened limbs, absent transverse flexural palmar creases, clinodactyly of the second and fifth finger, broad toes and nail dysplasia.

**Fig. 1:**
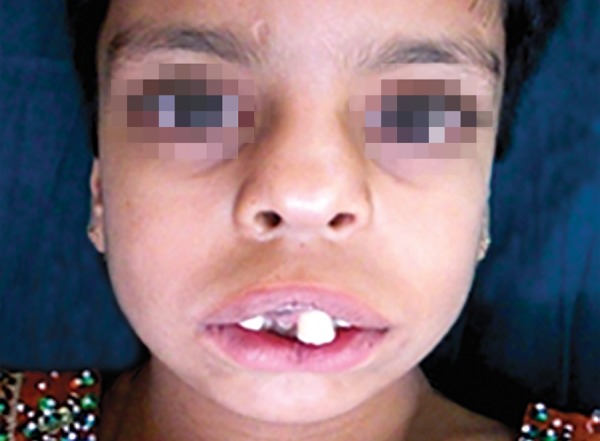
Extraoral view exhibiting typical orofacial features

Extraoral examination (Fig. 1) revealed macrocephaly, frontal bossing, hypertelorism, prominent eyes, down-slanting palpebral fissures, broad nasal bridge, upturned nose, long philtrum, deficient malar prominence depicting midfacial hypoplasia and mandibular retrognathism.

Oral features included triangular shaped mouth with downturned angles which exposed the maxillary incisors and the gums and midline indentation of upper and lower lip.

**Figs 2A to C:**
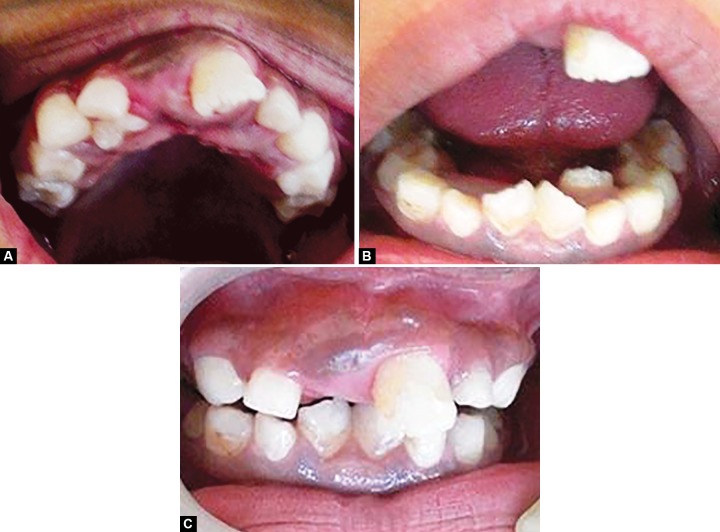
(A) Hypertrophy of maxillary ridge (B) Hypertrophy of mandibular alveolar ridge and (C) Intraoral view

Intraoral examination (Figs 2A to C) revealed high arched palate, narrowed and V-shaped mandibular arches, maxillary and mandibular alveolar ridge hypertrophy and short lingual frenulum depicting partial ankyloglossia. Teeth present were 11, 52, 53, 54, 55, 16, 21, 62, 63, 64, 26, 31, 32, 72, 73, 74, 75, 41, 82, 83, 84, 85. Palatally erupting 12, over retained 52, 62, 72, 82, lingually erupting 32, 42, mesiolingually rotated 31, 41.

**Fig. 3:**
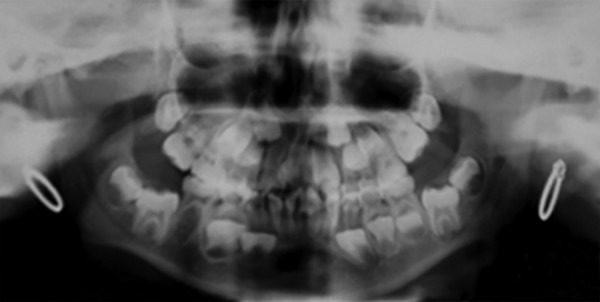
Orthopantomography revealing erupting 11, 22, 36, 46

**Figs 4A to C:**
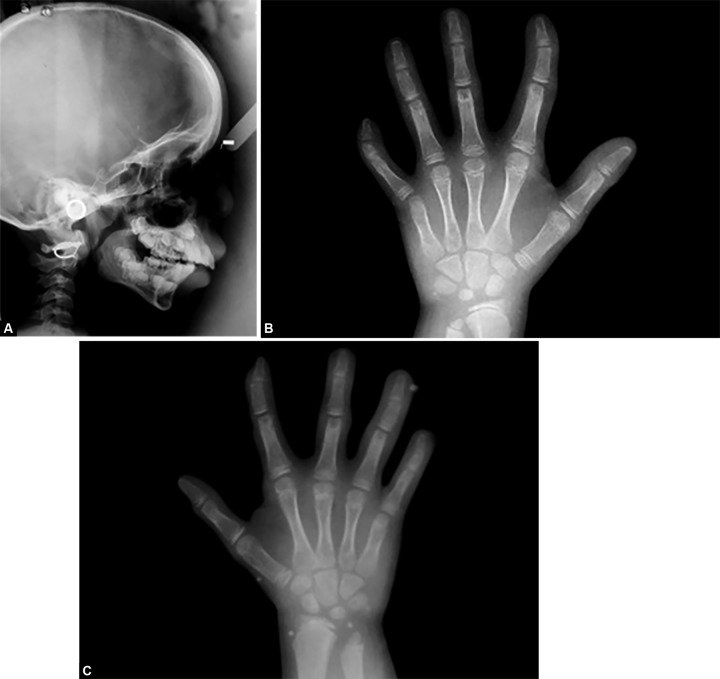
(A) Lateral cephalograph revealing midfacial hypoplasia and mandibular retrognathism, (B) Handwrist radiograph of the right hand showing clinodactyly of the second and fifth finger and (C) Handwrist radiograph of the left hand showing clinodactyly of the second and fifth finger

Orthopantomography (Fig. 3) revealed erupting 11, 22, 36, 46. Lateral cephalograph (Fig. 4A) depicts midfacial hypoplasia and mandibular micrognathism. Normal skeletal maturity was assessed through hand wrist graphs (Figs 4B and C) which exhibited clinodactyly of fifth finger. The patient was referred to the department of Pediatrics for further evaluation where she was identified as a case of Robinow syndrome. Patient underwent full body survey. Ultrasonography of abdomen and ECG was taken which were normal. Full skeletal survey revealed vertebral deformities which were characteristic of recessive form of the syndrome. A final diagnosis of an autosomal recessive Robinow syndrome was given. Anesthetic interventions were alerted to be preceded with precautions.

The patient underwent oral prophylaxis and full mouth fluoride application. Patient is under follow-up for further orthodontic treatment.

## DISCUSSION

The Robinow syndrome is a rare form of mesomelic dwarfism which is characterized by dysmorphic features but known biochemical or cytogenetic markers.^1,2^ It was Robinow et al in 1969 who reported several individuals in a family exhibiting mesomelic limb shortening, hypertelorism and hypoplastic genitalia.^3-6^ Based on the initial pedigree, this syndrome was attributed to be an autosomal dominant inherited syndrome [Online Mendelian Inheritance in Man (OMIM) 180700] with high penetrance.^4,5,7^ In 1978, a new autosomal recessive syndrome associating mesomelic limb shortening to a pattern of facial characteristics, hypoplastic genitalia and costovertebral defects was put forth by Wadia et al known with an acronym COVESDEM (costovertebral segmentation defects with mesomelia), later recognized as a variant of RS with an alternative inheritance mode (OMIM 238310).^4,8^

The gene for the autosomal recessive Robinow syndrome was mapped and first localized to chromosome 9q22. The tyrosine kinase-like orphan receptor 2, ROR2 gene was also located in this region and heterozygous mutations in ROR2 is been linked to the autosomal dominant condition brachydactyly type B. ROR2 is a member of the ROR family of receptor tyrosine kinases (RTKs).^4,5,7,9,10^

The autosomal recessive form of Robinow syndrome (MIM 268310) which is characterized by severe skeletal, vertebral and craniofacial defects usually caused by loss-of-function of ROR2 and rib fusions. Heterozygous mutations in ROR2 results in brachydactyly type B associated with terminal deficiency of fingers and toes. It has been suggested that ROR2 is necessary for proper proliferation, maturation, motility and function of chondrocytes resulting in normal formation and ossification which gives rise to the overall skeletal size.^4,9^ The distal mutations rank high in severity than the proximal mutations.^11^

The phenotypes of the dominant and recessive forms that overlap in Robinow syndrome implicate WNT5A as a ligand for ROR2. WNT signaling pathway activates cell proliferation and cell fate change during development. It has been analyzed that the mutations in WNT5A are linked to the autosomal dominant pattern of Robinow syndrome and this attributes to its role for human craniofacial and skeletal development.^5^

The facial features presented in early childhood are distinct with hypertelorism, midfacial hypoplasia, short upturned nose with flat nasal bridge, broad and prominent forehead. Robinow idealizes these characteristics to a fetal face by emphasizing the relatively small face, laterally displaced eyes and forward pointing alae nasi. The appearance is progressively dynamic and becomes less marked with time. The significant oral features which are distinctive and abet in the diagnosis are tented or downturned upper lip to an inverted V appearance with indentation in the center. This causes the exposure of the incisors and upper gum. Midline clefting of the lower lip can also occur. Gingival hypertrophy may also be a presenting feature from birth. Ankyloglossia is also a feature and when prominent, may appear as a bifid tongue. Malocclusion in the form of crowding and irregular teeth may be seen in primary and secondary dentition. The eye appears prominent due to the deficiency of the lower eyelid and resemble an exophthalmos but varies since they do not protrude from the orbit. Low set ears with or without deformation of the pinna is yet another feature. Seldom occurrence of midline capillary hemangioma may be noted.^4,6,12^

In most another skeletal dysplasias with dwarfism, the limb shortening is rhizomelic. In Robinow syndrome the limb shortening is mesomelic or acromesomelic and is more striking than the shortening in the leg. Made-lung deformity has been reported. Brachydactyly with shortening of the distal phalanx and nail hypoplasia or dystrophy, displaced or bifid thumb, incomplete cutaneous syndactyly of hands and feet, reduced stature, mac-rocephaly are features of this syndrome. The dermato-glyphic pattern unveils the underlying maldevelopment of the hands with absent interphalangeal creases, bilateral transverse creases, proximal flexion crease of the little fingers and a hypothenar whorl pattern.^1,4,6,12^

Such patients are perhaps prone to infection.^13^ Seldom congenital heart abnormalities like atrial septal defect, ventricular septal defect, coarctation of the aorta, tetralogy of Fallot, severe pulmonary stenosis or atresia, and tricuspid atresia and right ventricular outflow obstruction has been reported, commonest abnormality being pulmonary stenosis or atresia. These cardiac defects are probably the major cause of mortality in this syndrome in the first years of life.^4,8,14^

Endocrine abnormalities along with genital abnormalities may be noted at birth and in some cases cause concern regarding gender assignment at birth. In females, the anatomical defect is seldom obvious with reduced clitoral size and hypoplasia of the labia minora. In males, micropenis can occur with normal scrotum and testes.^15,16^

A salient feature is the splitting of one or more distal phalanges seen in handwrist radiographs and may show fusion of the phalanges and fusion of the carpal bones. Kyphoscoliosis and chest deformity is due to widespread fusion of thoracic vertebrae with frequent hemivertebrae. There may also be fusion of the ribs.^4^ Costovertebral segmentation defect could occur in severity in the thoracic part of the column and involving all vertebrae exhibiting as butterfly vertebrae, hemivertebra and vertebral fusion.^17^

The characteristic clinical dysmorphic features paves an easy way to diagnose recessive RS, in contrast to the dominant RS, in the lack of mesomelia and therefore other syndromes that go along with similar facial dys-morphic features (especially hypertelorism) and genital hypoplasia, such as Aarskog syndrome (MIM305400) and Opitz G syndrome (MIM300000) can be thought of as a differential diagnosis.^8^

Robinow syndrome has an incidence of 1 : 500 000 and a 1 : 1 male-to-female ratio. Despite its rare occurrence, the prevalence is lower, due to infant or early childhood mortality.^6,7,9^ Hence, a detailed cardiac assessment is necessary especially if the parents are among highly consanguineous population.^4,14^ Some degree of mental retardation may be exhibited in few (20%), but in majority, the intelligence is normal and adequate sexual functioning and reproduction can occur which is a good prognostic indicator.^6,18^
